# Ensemble perception of duration and size

**DOI:** 10.3758/s13414-025-03125-3

**Published:** 2025-08-12

**Authors:** Daniel Bratzke, Ruben Ellinghaus

**Affiliations:** 1 Department of Psychology, University of Bremen, Bremen, Germany; 2 Department of General Psychology: Judgment, Decision Making, Action, Faculty of Psychology, University of Hagen (FernUniversität in Hagen), Hagen, Germany

**Keywords:** Ensemble encoding, Time perception, Size perception

## Abstract

This study investigated ensemble perception of temporal (duration) as well as spatial (size) information with simultaneously and sequentially presented ensembles of different set size. The results showed that summary statistics can be extracted from temporal ensembles as well as from size ensembles, irrespective of whether the ensembles are presented simultaneously or sequentially, demonstrating the domain generality of ensemble perception. Nevertheless, the results also indicate clear domain-specific differences between the two dimensions. For simultaneous ensembles, mean estimates increased with set size for duration ensembles and decreased with set size for size ensembles, suggesting a possible bias by dimension-specific features; in the case of duration by the interoffset intervals, and in the case of size by the overall ensemble size. For sequential ensembles, there was a recency effect for size stimuli but not for duration stimuli, suggesting that the information is integrated for the two dimensions differently. For example, participants might rely on an internal prior formed by memory mixing more strongly in the case of relatively noisy representations of temporal information.

## Introduction

In one of the first studies on ensemble encoding of stimulus features, Ariely (2001; but see also Miller & Sheldon, 1969) asked what information people gather when they are confronted with a set of stimuli and observed that the visual system reflected the statistical properties of the overall set rather than those of the individual stimuli within the set. More specifically, in Ariely’s study, participants first saw a set of dots of different sizes and a subsequent test dot, and had to indicate whether the test dot appeared in the set or whether the test dot was smaller or larger than the mean size of all dots in the set. The results showed that member identification performance was rather poor, whereas performance in the mean discrimination task was relatively good. Subsequent studies have shown that the visual system computes accurate ensemble representations not only for the mean size, but also for other features such as brightness, orientation, and location (Alvarez, 2011). The ability to extract summary statistics from stimulus ensembles is not restricted to basic visual features but can also be observed for emotion, gender, face identity, and numbers (Alvarez, 2011).

Furthermore, summary statistics can be extracted not only from simultaneously, but also from sequentially presented stimulus ensembles (Hubert-Wallander & Boynton, 2015). Functionally, summary statistics (e.g., mean and variability) of stimulus ensembles reduce redundancy in highly structured environments (Alvarez, 2011) and may thus preserve just the information needed to form a global percept, identify candidate locations of interest, and navigate in this world (Ariely, 2001). Summary statistics may be especially useful in social environments because they carry emergent and social information at the level of the group (Whitney & Yamanashi Leib, 2018).

### Time: A special dimension

The dimension of time, just like space, is fundamental to the physical world (e.g., Maudlin, 2012) and our intuitions about it (e.g., Hatfield, 2006), as well as our perception (e.g., Kubovy & Van Valkenburg, 2001). The temporal domain, however, has long held a special status in the study of human cognition and perception. Unlike other perceptual dimensions commonly investigated in psychophysics (e.g., size, weight, brightness), time lacks a dedicated sensory system, as there is no direct physical stimulus for it (e.g., Grondin, 2010). Moreover, time is especially challenging to define physically (e.g., Callender, 2010) as well as psychologically (e.g., Zakay, 2016). These factors likely contribute to the enduring interest in comparing temporal and nontemporal stimuli across various perceptual tasks. As early as 1865, Ernst Mach observed that Weber’s Law does not apply to duration discrimination; instead, there exists a specific interval range of maximal discrimination sensitivity. While this finding suggests that temporal processing differs, at least to some extent, from other perceptual dimensions, later research revealed that some empirical laws once thought to be time specific are in fact domain general. For example, Vierordt (1868) found that reproduced durations tend to gravitate toward the overall mean of a stimulus series. However, Hollingworth later demonstrated that such central tendency biases extend beyond time perception, constituting a domain-general principle (Hollingworth, 1910). More recently, researchers have debated whether stimulus order effects on discrimination sensitivity differ between temporal and nontemporal stimuli (Ellinghaus et al., 2018, 2024), aiming to assess the generality of models of stimulus discrimination. Ultimately, the comparison of temporal and nontemporal stimuli serves as a powerful tool for identifying which aspects of cognitive processing are unique to temporal information and which reflect broader, domain-general principles. The present study therefore compared ensemble encoding of temporal (duration) and spatial (size) information.

### Integrated coding of temporal information

With respect to the question of how people encode temporal ensembles, there is ample evidence that temporal information is integrated over time to form internal references (for an overview, see Bausenhart et al., 2016), and timing of one interval is influenced by other temporally overlapping intervals (Brown & West, 1990; Bryce & Bratzke, 2016; Bryce et al., 2015; Van Rijn & Taatgen, 2008; for a general overview of time perception and timing methods, see Grondin, 2010). Although direct research into the perception or encoding of temporal ensembles and their related summary statistics is relatively limited to date (Curtis & Rule, 1977; Otsuka et al., 2025; Ren et al., 2020; Schweickert et al., 2014; Wearden & Jones, 2007), all existing studies suggest that people are able to extract summary statistics from ensembles of temporal intervals, just as they are able to extract them from spatial or other ensembles. For example, Ren et al. (2020) employed ensemble encoding of temporal intervals to investigate the metrical structure of the subjective timeline (see also Wearden & Jones, 2007). In each trial, participants had to indicate whether a test interval was shorter or longer than the mean duration of a series of temporal intervals (demarcated by short auditory beeps or brief flashes of a gray disk). The authors used an adaptive psychophysical procedure to assess the point of subjective equality (PSE) and the difference threshold (just noticeable difference, JND), reflecting the participant’s estimate of the mean duration and the precision of the estimate, respectively. The results revealed that PSEs were close to the objective geometric mean, consistent with a logarithmic representation of encoded time^1^ (similar to the logarithmic mental number line; e.g., Dehaene, 2003).

### Simultaneous and sequential ensemble encoding

Previous studies have shown that the precision of mean judgments is rather independent of the set size of the ensemble (e.g., Allik et al., 2013; Alvarez, 2011; Ariely, 2001), which has led some researchers to suggest that only a limited number of elements can be taken into account in the computation of the summary statistic (e.g., Allik et al., 2013). Whitney and Yamanashi Leib (2018) showed that approximately the square root of the number of displayed objects is integrated in ensemble perception, irrespective of whether the ensembles are presented simultaneously or sequentially. Accordingly, it has been suggested that ensemble perception generally operates similarly for these two presentation modes, assuming that even for simultaneous ensembles information is effectively sampled serially in time through shifts in visual attention (Hubert-Wallander & Boynton, 2015). Nevertheless, the encoding of sequential ensembles might be more prone to the serial dependence effect, that is that perception of a currently presented stimulus is biased towards previously presented stimuli (for reviews, see Cicchini et al., 2024; Sadibolova & Terhune, 2022). Furthermore, previous research with a sequential presentation of the ensemble has shown recency effects (i.e., stimuli at later positions in the processing had the strongest influence on mean estimates) for object size, facial expression, and motion direction, but a primacy effect for mean location, suggesting differences in ensemble encoding between different domains (Hubert-Wallander & Boynton, 2015). Thus, comparing encoding of sequential ensembles and the associated weighting of sequentially presented information between time and space has the potential to reveal further domain-specific characteristics of temporal ensemble encoding beyond the simultaneous encoding situation.

### Present investigation

**Fig. 1:**
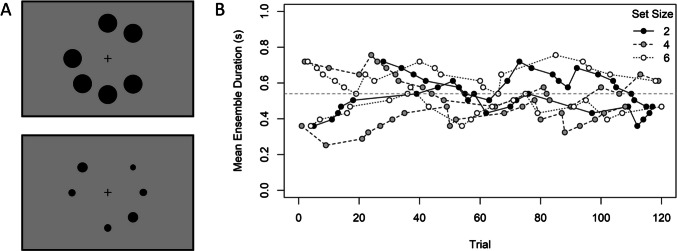
**A** Examples of stimulus ensembles in Experiment 1 (top) and Experiment 2 (bottom). In the simultaneous presentation mode, all stimuli appeared at once. In the sequential mode, the stimuli were presented one after each other, with an interstimulus interval of 500 ms. Each stimulus appeared randomly (without replacement) at one of the eight possible locations. **B** Example data of one participant (simultaneous ensembles with a standard duration of 540 ms). Each set size was tested with an upper and a lower run (starting with mean ensemble durations longer and shorter than the standard duration, respectively). Trials for the different set sizes (2, 4, and 6) were tested intermixed. The mean ensemble duration changed adaptively with the participant’s response (separately for each set size and run); it was decreased if the response was “longer” and increased if the response was “shorter”

In the present study, we investigated ensemble encoding in two different experiments (one with duration and one with size ensembles), with simultaneous and sequential ensembles of different set size (2, 4, and 6) and different stimulus magnitude (duration: 540 vs. 1,080 ms, size: 1 vs. 2 cm). To assess the subjective mean of the ensemble and its precision, we employed an adaptive single stimulus procedure following the weighted up–down method (see Kaernbach, 1991). Before each test phase, participants were presented with a standard dot of a certain duration (or size). In each trial of the test phase, they were presented with an ensemble of circularly arranged dots of different durations (or sizes; see Fig. 1A) and had to indicate whether the mean of the ensemble was shorter (smaller) or longer (larger) than the standard duration (or standard size). Depending on the participant’s response, the mean of the ensemble in the next trial was increased (after a “shorter”/“smaller” response) or decreased (after a “longer”/“larger” response). Applying two runs (an upper and a lower run, which started with ensemble means that were longer and shorter than the standard duration, respectively), this procedure allowed us to track the subjective mean of the ensemble (i.e., the PSE) and its precision (i.e., the Weber fraction, WF = JND/PSE), separately for each standard magnitude and set size (see Fig. 1B). In the example of duration, a PSE larger than the standard duration indicates an underestimation of the ensemble mean duration (i.e., the mean duration of the ensemble must be longer than the standard duration for both to be perceived as equally long), while a PSE smaller than the standard duration indicates an overestimation of the ensemble mean duration (i.e., the mean duration of the ensemble must be shorter than the standard duration for both to be perceived as equally long).

### Simulation-based predictions

In order to assess the predictions regarding the effects of our experimental manipulations (i.e., set size and standard magnitude) as well as of the subsampling and serial dependence hypotheses, we ran simulations according to the basic design of our experiments and used duration as an exemplar target dimension for illustration. As in the experiments, in each trial the ensemble represented a normal distribution with a mean corresponding to the mean ensemble magnitude and a standard deviation proportional to the mean. To account for the scalar property of timing (i.e., increasing variability with increasing duration; e.g., Wearden & Lejeune, 2008; see also Ogden et al., 2021, for scalar variability in time, numerosity, and length) in the simulations, Gaussian noise with a variance proportional to the stimulus duration (*sd* = stimulus_duration*/*5) was added to each stimulus duration. Twenty trials were simulated for each set size and run (as in our experiments; see also Fig. 1B), and the data of *N* = 1,000 participants were simulated. In the simulation with serial dependence, each stimulus duration within an ensemble was averaged (arithmetic mean) with the duration of the preceding stimulus. For subsampling, we considered the cases that only two, four, or all stimuli (irrespective of set size) are sampled from the ensemble.

**Fig. 2:**
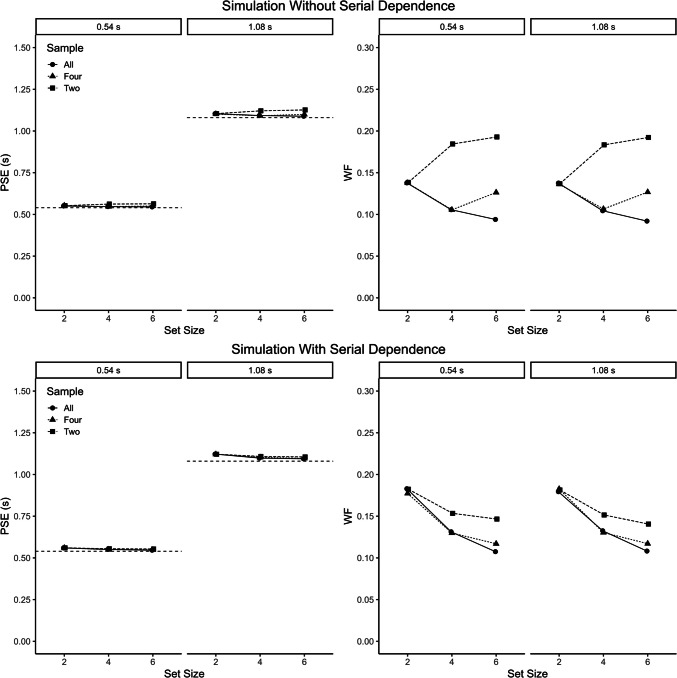
Predicted point of subjective equality (PSE, left panels) and Weber fraction (WF, right panels) as a function of set size, sample, and standard duration. The upper graphs show simulation results without serial dependence, the lower graphs with serial dependence. Dashed horizontal lines indicate the standard durations

Figure 2 shows the predicted result patterns according to these simulations. In order to assess the influence of subsampling it is useful to first consider the case without serial dependence (see upper part of Fig. 2). If participants sample all stimuli from the ensemble, the PSE should be fairly constant across set sizes, and the WF should decrease with increasing set size. If participants sample only a subset of stimuli from the ensemble, the PSE should again be rather unaffected by set size; however, the WF should increase as soon as the set size exceeds the sampled subset. With serial dependence (which is especially likely for sequential ensembles), WFs should be on average larger than without serial dependence, and WFs should also decrease more strongly with increasing set size with than without serial dependence (compare right lower with right upper panel of Fig. 2). To additionally investigate possible differences in the weighting of information from different stimuli in the ensemble (primacy or recency effects), we used logistic regression analyses (similar to Hubert-Wallander & Boynton, 2015).

## Experiment 1: Ensemble perception of duration

### Method

#### Participants

A sample of 52 participants recruited from the student pool at the University of Hagen was tested online and compensated with course credit. This sample size was chosen to achieve a statistical power of 0.80 with an alpha of 0.05 for a large effect size of η_p_^2^ = 0.14. The data of three participants were excluded from analyses because no PSE could be estimated from their data at least in one experimental condition (they had no response reversals in the adaptive procedure) and replaced by the data of new participants. The mean age of the final sample was 31.5 years (*SD* = 10.4, 33 women, 15 men, two nonbinary, two participants indicated female in one session and male in the other).

#### Apparatus and stimuli

The experiment was an online experiment and run on the participant’s individual computer. It was created in PsychoPy (Peirce et al., 2019) and hosted by Pavlovia (https://pavlovia.org). The PsychoPy code ScreenScale (Morys-Carter, 2021) was used to adjust the screen scale on individual computers. Thus, the reported size measures assume that participants adjusted their screens according to these instructions. All visual stimuli were presented on a gray background with a centrally positioned black fixation cross (width and height: 1 cm). The stimuli were black filled circles (2.5 cm in diameter), which could appear in eight circularly arranged positions around the central fixation cross (0, 45, 90, 135, 180, 225, 270, and 315°), with a distance of the center of each circle from the center of the monitor of 5 cm. For each trial, the durations of the individual circles were randomly drawn from a normal distribution with a mean of *ensemble_duration* and a standard deviation of *ensemble_duration*/4. These durations were then standardized that their sample mean and standard deviation corresponded to *ensemble_duration* and *ensemble_duration*/4, respectively.

#### Overall procedure

Each participant took part in two experimental sessions (simultaneous vs. sequential, counterbalanced across participants), with no more than 2 days between sessions. Each session started with a tutorial demo of two exemplary trials (with Set Sizes 2 and 4), which explained the concept of the average of the durations (explicitly presenting the duration of each stimulus and the mean duration of the ensemble). The subsequent experiment only started if the participant provided the correct answer to a test item at the end of the tutorial (e.g., “If the durations of two presented circles were 1 and 2 s, what would be the mean duration: 1.5 or 2.5 s?”). In case of an incorrect response, the tutorial started again. Participants could also choose to view the tutorial again if they provided the correct answer. The main experiment was divided into two halves, one for each standard duration (540 vs. 1,080 ms, with random order). Each half started with a learning phase, in which participants were presented with the standard duration, followed by a testing phase (a short practice and an experimental block), including the adaptive psychophysical procedure.

#### Task and adaptive procedure

At the beginning of each experimental half, participants saw a standard duration five times. The presentation of each standard duration was initiated with the space key. After pressing the key, the fixation cross appeared for 750 ms, and after another 250 ms, a central filled circle appeared for one of two possible standard durations (*standard_duration*: 540 vs. 1,080 ms). In the following testing phase, participants had to indicate whether the average duration of a circle ensemble was shorter (with the “X” key) or longer (with the “M” key) than the initially presented standard duration. Each testing phase started with a practice block of six trials in random order, with two trials from each set size (2, 4, or 6), one with a shorter mean duration than *standard_duration* (*standard_duration − standard_duration*/3) and one with a longer mean duration (*standard_duration* + *standard_duration*/3). In the practice block, participants received feedback about the correctness of their response; no feedback was provided in the experimental block.

In each experimental block, there were two adaptive runs (upper and lower) for each set size (each containing 20 trials), targeting the 25th and 75th percentile of the psychometric function, respectively. The two runs started with mean durations, *ensemble_duration* = *standard_duration* ± *standard_duration*/3, respectively. Within the two runs *ensemble_duration* changed adaptively depending on the participant’s response. The step size of these changes was 36 ms for the short and 72 ms for the long standard duration. In the upper run, *ensemble_duration* was reduced by one step size, if the participant indicated that *ensemble_duration* was longer than *standard_duration*, and increased by three step sizes, if they indicated that it was shorter. In the lower run, *ensemble_duration* was increased by one step size, if the participant indicated that *ensemble_duration* was shorter than *standard_duration*, and reduced by three step sizes, if they indicated that it was longer. The trials of the two runs within each experimental block were presented intermixed in random order. There were 20 trials for each combination of run (high vs. low) and set size (2, 4, and 6), yielding 120 trials per experimental block. Thus, each participant performed 480 trials in total (120 trials per standard duration and presentation mode).

#### Simultaneous and sequential trial procedure

In each experimental trial, first the fixation cross appeared for 750 ms. After another 250 ms, the test circles were presented. In trials with the simultaneous presentation mode, all circles appeared simultaneously (but had asynchronous offsets). In trials with the sequential presentation mode, the circles were presented one after the other in random order with an interstimulus interval of 500 ms.

#### Data analysis

We used the PSE as a measure of the participant’s estimate of the ensemble mean and the Weber fraction (WF = JND/PSE) as a measure of the estimate’s precision. For the calculation of individual PSEs, first the reversal points within the runs (i.e., when the participants changed their response), separately for each presentation mode, target duration and set size, were identified. Then, the mean duration of the ensembles that corresponded to these reversal points were averaged, yielding the 75th (upper run) and 25th (lower run) percentile of the psychometric function. Finally, these means from the upper and lower runs were averaged to obtain the PSEs (i.e., the 50th percentile of the psychometric function). For the calculation of individual WFs, first the JND was calculated as half of the absolute difference between the reversal averages for the upper and lower run for each condition (see Ellinghaus et al., 2018; Luce & Galanter, 1963). Then the WF was calculated by dividing the JND by the PSE. Logistic regression analyses were performed to assess the relative influence of each member of the ensemble on the estimates (see Hubert-Wallander & Boynton, 2015).

### Results

**Fig. 3:**
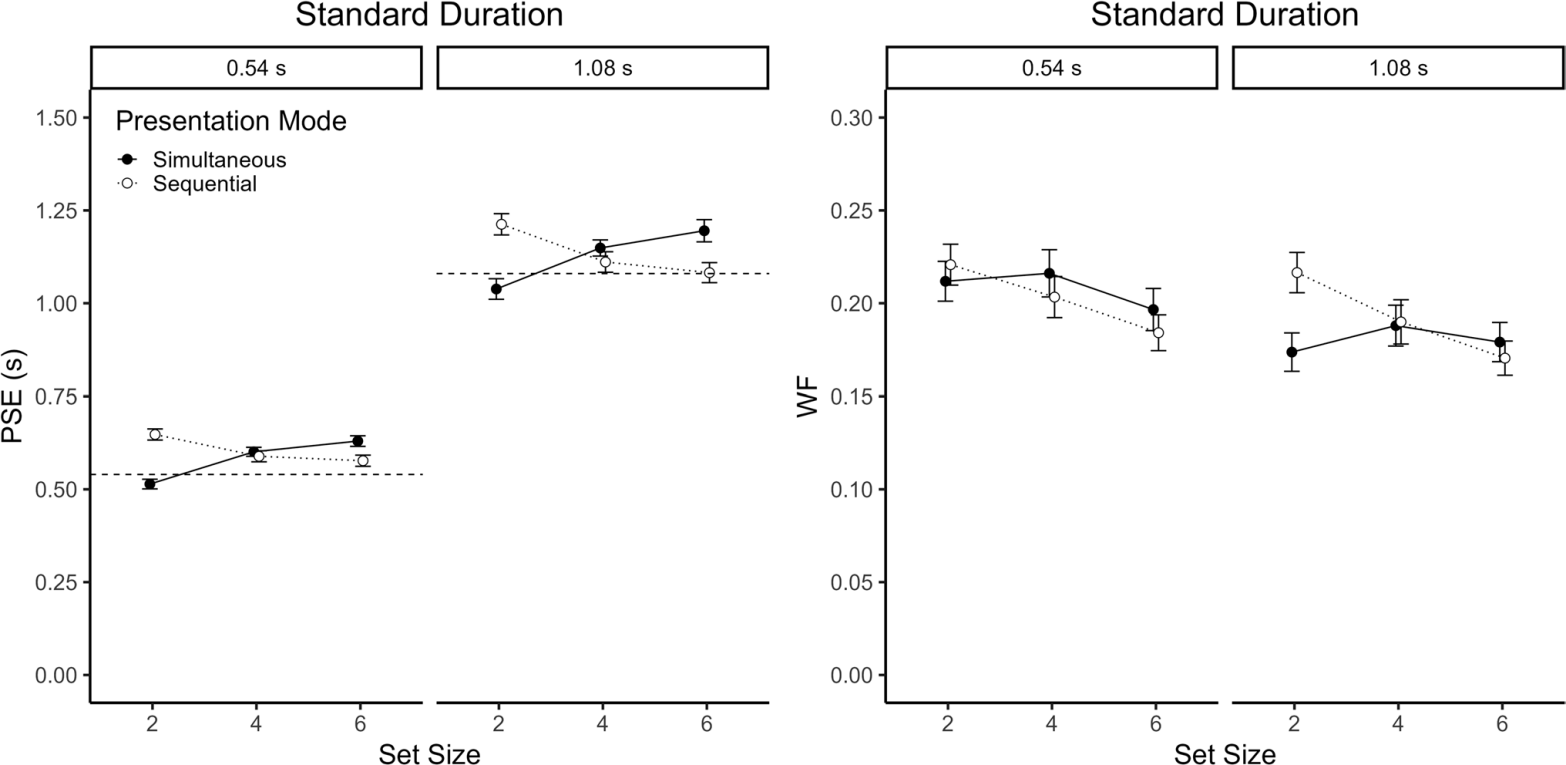
Point of subjective equality (PSE, left panel) and Weber fraction (WF, right panel) as a function of set size, presentation mode and standard duration in Experiment 1. Dashed horizontal lines indicate the standard durations. Error bars represent ± 1 within-subject *SE* (Morey, 2008)

Figure 3 shows PSE (left panel) and WF (right panel) as a function of standard duration, set size and presentation mode. Separate analyses of variance (ANOVAs) with the within-subject factors standard duration, set size, and presentation mode were conducted for PSE and WF. The Greenhouse–Geisser correction was used to adjust *p* values where appropriate.

#### Point of subjective equality

The ANOVA on PSE showed a main effect of standard duration, *F*(1, 51) = 889.79, *p* < 0.001, η_p_^2^ = 0.95, but no main effect of size, *F*(2, 102) = 1.30, *p* = 0.270, η_p_^2^ = 0.02, and presentation mode, *F*(1, 51) = 0.64, *p* = 0.499, η_p_^2^ = 0.01. PSE was 0.59 s for the short and 1.13 s for the long standard duration. Thus, the mean duration of the ensemble was slightly underestimated for both standard durations. As can be seen in Fig. 3, there was a clear interaction between set size and presentation mode, *F*(2, 102) = 68.87, *p* < 0.001, η_p_^2^ = 0.57. PSE increased with set size in the simultaneous mode (0.78, 0.87, 0.91 s; *p* < 0.001), but decreased with set size in the sequential mode (0.93, 0.85, 0.83 s; *p* < 0.001). The significant three-way interaction, *F*(2*,* 102) = 5*.*07*, p* = 0.010, η_p_^2^ = 0.09, confirmed that this interaction pattern was more pronounced for the long than for the short standard duration (see Fig. 3). All other two-way interactions were not significant, *F* values < 1.

#### Weber fraction

The ANOVA on WF showed significant main effects of standard duration, *F*(1, 51) = 5.17, *p* = 0.027, η_p_^2^ = 0.09, and set size, *F*(2, 102) = 6.61, *p* = 0.002, η_p_^2^ = 0.11, but no main effect of presentation mode, *F*(1, 51) = 0.12, *p* = 0.733, η_p_^2^ < 0.01. The WF was larger for the small (0.21) than for the large (0.19) standard duration, and decreased with increasing set size (0.21, 0.20, 0.18). There was also a significant interaction between set size and presentation mode, *F*(2, 102) = 3.94, *p* = 0.023, η_p_^2^ = 0.07. As can be seen in Fig. 3, the WF showed a clear decrease with increasing set size for sequential ensembles (0.22, 0.20, 0.18; *p* < 0.001), whereas the WF was relatively unaffected by set size for simultaneous ensembles (0.19, 0.20, 0.19; *p* = 0.325). All other interactions were not significant, *p* values > 0.185.

#### Logistic regression

**Fig. 4:**
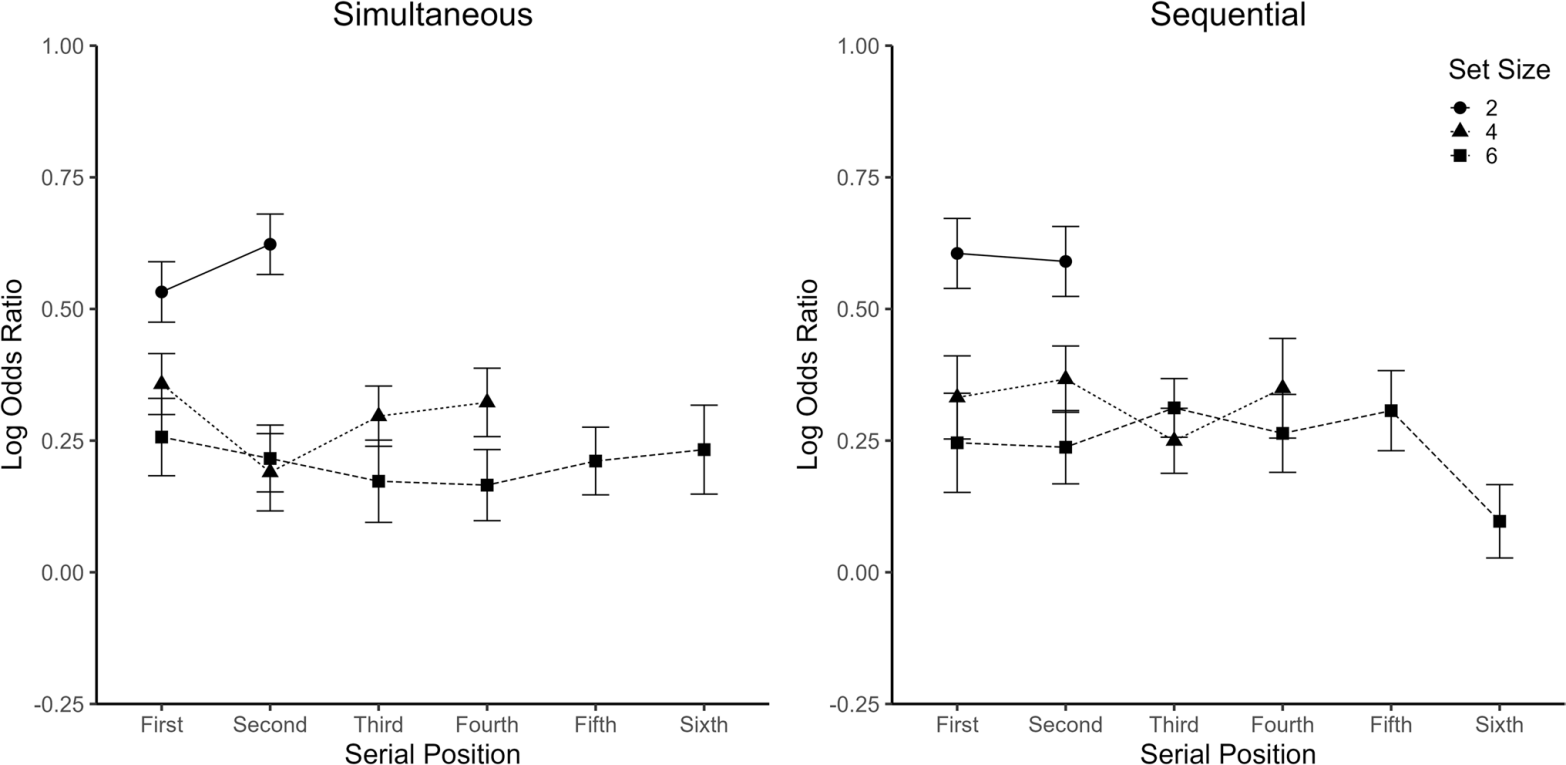
Results of the logistic regression in Experiment 1. Log odds ratio as a function of serial position and set size, separately for the two presentation modes. Error bars represent ± 1 within-subject *SE* (Morey, 2008). Note that for simultaneous ensembles, serial position is essentially a dummy variable as all dots were presented at the same time at random positions

For the logistic regression, data were collapsed across the two standard durations. Separate logistic regressions were conducted for each set size and presentation mode, with the duration of each dot (*z* standardized) at each serial position (from first to sixth) in the ensemble as predictor and the participant’s response (“shorter” vs. “longer,” coded as 0 vs. 1) as outcome. Note that for simultaneous ensembles, the position variable is essentially a dummy variable as all dots were presented at the same time at random positions. Figure 4 shows log odds ratios as a function of serial position, set size, and presentation mode. Separate ANOVAs for the three set sizes with the within-subject factors serial position and presentation mode showed no significant main or interaction effect, *p* values > 0.204.

### Discussion

Estimates of the mean (i.e., PSEs) were close to the standard duration, indicating that participants were able to extract the summary statistic from the temporal ensemble. Nevertheless, the ensemble means were slightly underestimated (i.e., PSEs were larger than the objective mean). Moreover, the two presentation modes showed differential results regarding the influence of set size, with none of the results matching the prediction of rather constant PSEs across set sizes (see Fig. 2). While PSEs increased with set size for simultaneous ensembles, this trend was reversed when the stimuli were presented sequentially. This means that participants estimated the mean duration to be even shorter for larger set sizes in the case of simultaneous ensembles, but to be relatively longer (i.e., more veridical) for sequential ensembles. Moreover, this interaction pattern was stronger for the long than for the short standard duration. In the simultaneous mode, participants might have been biased by the temporal dynamic of the stimulus offsets in the simultaneous mode. Specifically, in this mode all stimuli appeared at the same time but had different temporal offsets. As a consequence, the rate of stimulus offsets increased with set size (i.e., the interval between individual offsets decreased). The relatively shorter interoffset intervals for larger set sizes might have induced a tendency to either perceive the mean to be shorter or choose the response “shorter.” In the case of sequential ensembles, the overall longer total trial duration with increasing set size might have induced a bias towards responding “longer,” leading to smaller PSEs with increasing set size.

The WFs (around 0.2) were of similar size as previously observed WFs for visual duration discrimination employing an adaptive procedure (e.g., Ellinghaus et al., 2018). The results showed that the precision of the estimate increased with set size for sequential ensembles, whereas it was rather constant across set sizes for simultaneous ones. This pattern is consistent with the hypothesis that there is a stronger sequential assimilation of the current stimulation towards previous stimuli (i.e., a stronger serial dependence effect) for sequential than simultaneous ensembles. As WFs did not increase with set size for either simultaneous or sequential ensembles, there was no evidence that participants sampled only a subset of stimuli for the calculation of the ensemble means.

## Experiment 2: Ensemble perception of size

Experiment 2 used the same general method as Experiment 1, but employed spatial rather than temporal ensembles. Experiment 2 thus allowed us to directly compare the results of Experiment 1 with the corresponding results regarding stimulus size using the same psychophysical approach.

### Method

#### Participants

A new sample of 52 participants recruited from the student pool at the University of Hagen was tested online and compensated with course credit. The data of three participants were excluded from analyses because negative size values appeared in at least one of the trials for at least one of the dots in the ensemble and replaced by the data of new participants. The mean age of the final sample was 31.7 years (*SD* = 10.6, 31 women, 20 men, 0 nonbinary, one participant indicated female in one session and male in the other).

#### Stimuli and procedure

The stimuli and procedure were the same as in Experiment 1, with the exceptions that all stimuli were presented with a constant duration of 500 ms and the standard sizes (diameter) of the dots were 1 and 2 cm. As in Experiment 1, the step sizes in the adaptive procedure were *standard_size*/15 (i.e., 66.7 mm for the small standard and 133.3 mm for the large standard).

### Results

**Fig. 5:**
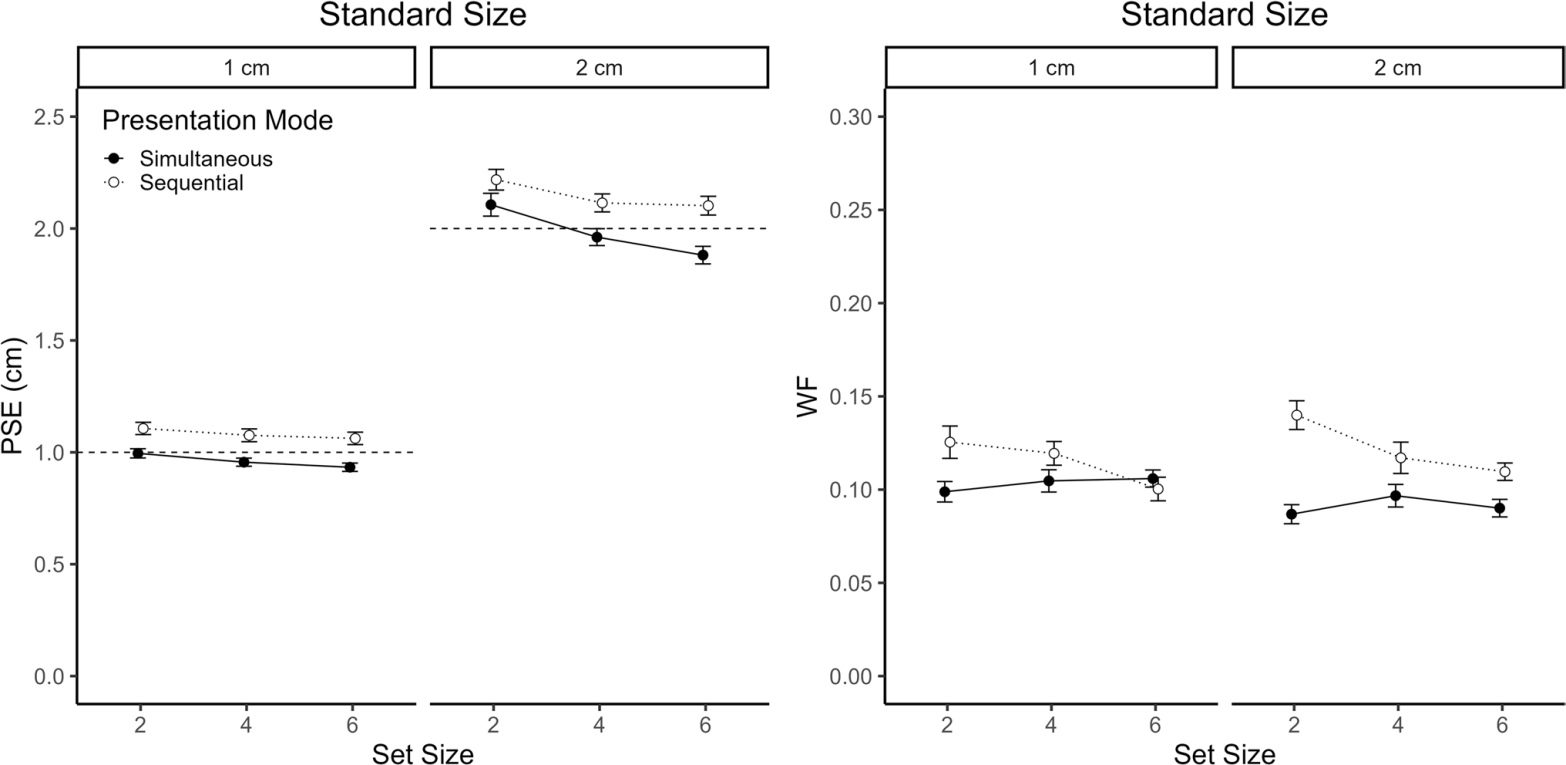
Point of subjective equality (PSE, left panel) and Weber fraction (WF, right panel) as a function of set size, presentation mode, and standard size in Experiment 2. Dashed horizontal lines indicate the standard sizes. Error bars represent ± 1 within-subject *SE* (Morey, 2008)

Figure 5 shows PSE (left panel) and WF (right panel) as a function of standard duration, set size and presentation mode. Separate ANOVAs with the within-subject factors standard duration, set size, and presentation mode were conducted for PSE and WF. The Greenhouse–Geisser correction was used to adjust *p* values where appropriate.

#### Point of subjective equality

In contrast to Experiment 1, the ANOVA on PSE showed main effects of all factors. PSE was smaller for the small (1.02 cm) than for the large standard size (2.06 cm), *F*(1, 51) = 816.81, *p* < 0.001, η_p_^2^ = 0.94, decreased with increasing set size (1.61, 1.53, 1.49 cm), *F* (2, 102) = 38.46, *p* < 0.001, η_p_^2^ = 0.43, and was smaller for simultaneous (1.47 cm) than for sequential (1.61 cm) ensembles, *F*(1, 51) = 16.35, *p* < 0.001, η_p_^2^ = 0.24. As in Experiment 1, there was a significant interaction between set size and presentation mode, *F*(2, 102) = 6.51, *p* = 0.005, η_p_^2^ = 0.11, and also a significant three-way interaction, *F*(2, 102) = 3.51, *p* = 0.042, η_p_^2^ = 0.06. However, as can be seen in Fig. 5, the interaction pattern was qualitatively different from the cross-interaction pattern in Experiment 1 (see Fig. 3). PSE decreased with increasing set size in both presentation modes (*p* values < 0.001), but the decrease was more pronounced for the large standard size, especially for simultaneous ensembles. Confirming this descriptive pattern, there was also a significant interaction between standard size and set size, *F*(2*,* 102) = 20*.*33*, p* < 0.001*,* η_p_^2^ = 0.28. The significant three-way interaction indicates that the interaction increased with standard size (see Fig. 5). The interaction between standard size and presentation mode was not significant, *F* < 1.

#### Weber fraction

Overall, the WFs were about half as large as in Experiment 1 (0.11 vs. 0.20). In contrast to Experiment 1, there was no significant main effect of standard size, *F*(1, 51) = 0.30, *p* = 0.587, η_p_^2^ = 0.01, but a significant one for presentation mode, *F*(1, 51) = 18.67, *p* < 0.001, η_p_^2^ = 0.27. As in Experiment 1, the main effect of set size was significant, *F*(2, 102) = 4.07, *p* = 0.020, η_p_^2^ = 0.07. The WF was smaller for simultaneous (0.10) than for sequential (0.12) ensembles, and slightly decreased with increasing set size (0.11, 0.11, 0.10). Presentation mode interacted significantly with standard size, *F*(2, 102) = 4.40, *p* = 0.041, η_p_^2^ = 0.08, and also with set size, *F*(2, 102) = 7.55, *p* = 0.001, η_p_^2^ = 0.13. The WF was slightly smaller for the large (0.09) than for the small standard (0.10) when the ensemble was presented simultaneously, but very similar (0.12 vs. 0.12) for sequential ensembles. Similar to Experiment 1, the WF clearly decreased with increasing set size for sequential ensembles (0.13, 0.12, 0.10; *p* < 0.001), but was rather constant across set sizes for simultaneous ensembles (0.09, 0.10, 0.10; *p* = 0.288). All other interactions were not significant, *p* values > 0.248.

#### Logistic regression

**Fig. 6:**
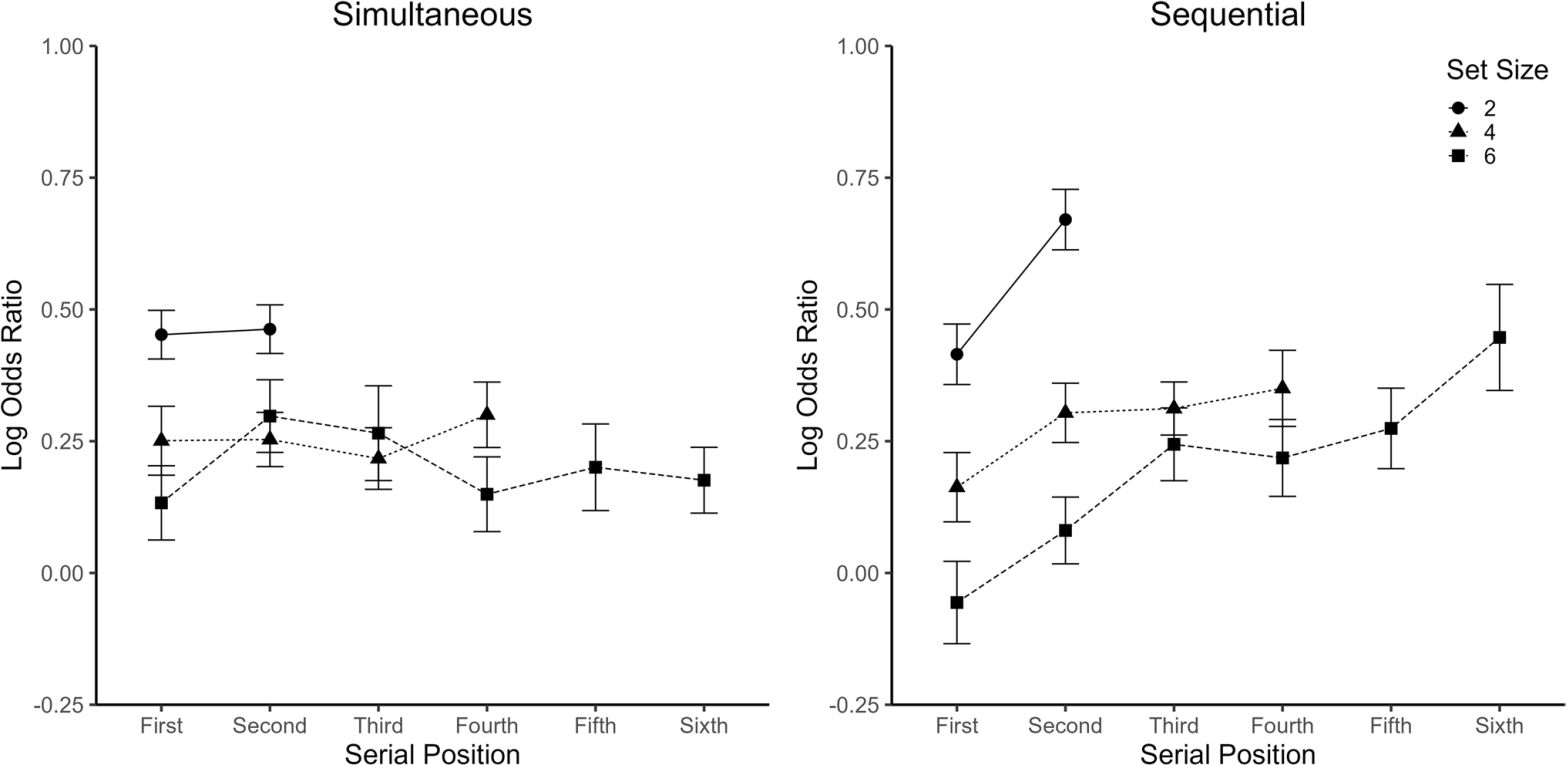
Results of the logistic regression in Experiment 2. Log odds ratio as a function of position and set size, separately for the two presentation modes. Error bars represent ± 1 within-subject *SE* (Morey, 2008). Note that for simultaneous ensembles, serial position is essentially a dummy variable as all dots were presented at the same time at random positions

The logistic regression analysis followed Experiment 1. Accordingly, data were collapsed across the two standard size conditions, and separate logistic regressions were conducted for each set size and presentation mode, with the size of each dot (*z* standardized) at each serial position (from first to sixth) in the ensemble as predictor. As shown in Fig. 6, log odd ratios for the simultaneous ensembles were rather unaffected by serial position; however, log odds ratios increased with serial position for the sequential ensembles. Accordingly, separate ANOVAs for the three set sizes showed significant main effects of serial position (*p* values < 0.014) and a significant interaction between serial position and presentation mode (*p* values < 0.024) for all set sizes except Set Size 4 (serial position: *p* = 0.292; serial position × presentation mode: *p* = 0.449).

### Discussion

The results of Experiment 2 differed in several aspects from the results with temporal ensembles in Experiment 1. The PSE results did not show the same cross-interaction pattern between presentation mode and set size as in Experiment 1. Essentially, PSEs were larger for sequential than for simultaneous ensembles (i.e., the mean was estimated to be smaller). Possibly, estimates of the mean size were biased by the overall size of the ensemble, which was more salient in the simultaneous mode. This explanation is also consistent with two other results—namely, that the PSE decreased with set size (i.e., ensembles with a larger set size were estimated as larger) and that the difference between the two modes increased with set size. Nevertheless, the PSE also showed a decrease with increasing set size for sequential ensembles (as in Experiment 1), which can be explained either also by a bias due to the increase of total ensemble size with increasing set size over time, or by cross-dimensional interference from the increasing total trial duration.

Although there were some differences in the WF result pattern in comparison with Experiment 1 (e.g., twice as small WFs), the interaction patterns were relatively similar. As in Experiment 1, the precision of the estimate increased with set size for sequential ensembles, whereas it was rather constant across set sizes for simultaneous ensembles. This is again consistent with the assumption of stronger serial dependence effects in sequential than simultaneous ensembles. Also, there was again no evidence for subsampling based on the WF results. In contrast to Experiment 1, logistic regressions showed a clear recency effect for the sequential presentation—that is, size judgments were more strongly influenced by stimuli at later positions within the presentation stream. This result is consistent with the previous observation of a recency effect for mean size using the more common adjustment method for measuring subjective ensemble summary statistics (Hubert-Wallander & Boynton, 2015). In summary, Experiment 2 showed clear differences to the results of Experiment 1 with temporal stimuli, while replicating the previously observed recency effect for sequential stimuli of different size.

### General discussion

The present study investigated ensemble perception of stimuli that varied in duration (Experiment 1) and size (Experiment 2). Overall, the results demonstrate that summary statistics can be extracted from ensembles of stimuli that vary in temporal features (see also Curtis & Rule, 1977; Otsuka et al., 2025; Ren et al., 2020; Schweickert et al., 2014; Wearden & Jones, 2007), as well as from ensembles of stimuli that vary in spatial features (e.g., Ariely, 2001), illustrating the domain-generality of ensemble encoding. Nevertheless the results also revealed domain-specific differences between the two stimulus dimensions. Specifically, mean estimates of duration and size ensembles showed different trends with increasing set size when the stimuli appeared at the same time. In the case of duration, mean estimates decreased with set size (i.e., PSEs increased), possibly biased by the temporal dynamic of the interstimulus offsets, which also decreased with set size; in the case of size, mean estimates increased with set size (i.e., PSEs decreased), possibly biased by the overall size of the ensemble, which was larger and presumably also more salient when set size increased. When the ensembles were presented sequentially, the PSE and WF result patterns were relatively similar for the two dimensions, showing a decrease (i.e., an increase in precision) with increasing set size. This PSE pattern can be explained by a bias due to the increasing total trial duration in the case of duration ensembles and total trial size in the case of size ensembles. Regression analyses also revealed differences between the two dimensions for this presentation mode, as there was a clear recency effect for size but not for duration ensembles. This suggests that stimuli at later positions in the presentation stream contributed more to the mean estimate than those at earlier positions, but this was only the case for size. Overall, there was no evidence that participants sampled only a subset of stimuli for the calculation of the ensemble means.

Recency effects in sequentially presented ensembles were previously reported by Hubert-Wallander and Boynton (2015) for size and also other stimulus dimensions, such as facial expression and motion direction. The present study extends their findings by providing regressions for different set sizes. Following previous considerations by these authors, the present finding of a significant recency effect even with only two members in the ensemble clearly argues against a major role of working memory capacity limitations in the recency effect. These results are rather in line with another explanation, which assumes that people adaptively make predictions about what the next item in a series will be based on what they have experienced before (Cheadle et al., 2014; Hubert-Wallander & Boynton, 2015). A related phenomenon is that perception is usually biased towards previously presented stimuli (serial dependence effect, e.g., Cicchini et al., 2024). A theoretical framework accounting for such temporal context effects is constituted by the internal reference model (Dyjas et al., 2012), according to which stimulus representations are continuously updated by mixing any current stimulus representation with the representation of past stimulus instances (for a meta-analytic overview of behavioral signatures indicating internal reference formation across both temporal and spatial stimulus aspects, see Ellinghaus et al., 2024). The present result that the mean estimates were less precise and additionally precision improved more strongly with set size for sequential than simultaneous ensembles is consistent with the idea that there is a stronger serial dependence in sequential than simultaneous ensembles (which may also be processed serially; see Hubert-Wallander & Boynton, 2015). The overall lower precision for sequential than simultaneous ensembles, however, could also be associated with the generally longer testing time for sequential than simultaneous ensembles, which might affect the memory representation of the standard as well as motivational factors.

Importantly, the present results did neither show a recency nor a primacy effect for duration (Hubert-Wallander & Boynton, 2015, observed a primacy effect for mean location). This indicates that all durations contributed to the mean estimate with similar weights. This difference between duration and size ensembles cannot be attributed to uncertainties about stimulus position as the procedure did not differ in this regard between the two dimensions. One could assume that, in addition to the number of stimuli, the total trial duration may be a critical factor for recency effects. Both factors, however, do not seem to play a role in the present context, as the lack of a recency effect in the case of duration as well as the occurrence of a recency effect in the case of size were largely independent of set size. Another potential factor might be the regularity of the temporal sequence. In the size experiment (Experiment 2), each stimulus was presented with a constant duration, whereas in the duration experiment (Experiment 1), the duration changed adaptively from trial to trial. Possibly, the irregular temporal sequence in Experiment 1 suggested to the participants that each stimulus was equally important for the calculation of the mean, or helped participants to stay focused on each stimulus. Another possibility is that the representation of temporal information is less accurate and stable than that of size information (WFs were twice as large for duration as for size). If the current stimulus representation is rather noisy, it might be weighted less strongly when integrated with the internal reference from past stimuli (see Ellinghaus et al., 2018) or deviate less from the predictions derived from previous stimulation (see also Hubert-Wallander & Boynton, 2015). How exactly the information is integrated in sequential duration and size ensembles (as well as other ensembles) clearly remains an open question for future research.

The present study employed an adaptive single stimulus procedure to assess the mean estimate of the ensemble as well as its precision. This procedure is yet rather uncommon in ensemble perception research (but see Ren et al., 2020, who also used an adaptive procedure with temporal stimuli). This raises the question of its validity and the generalizability of the obtained results. For example, one could suspect that the single stimulus method with a presentation of the standard stimulus only before the test phase does not establish a sufficiently stable representation of the standard magnitude. Previous research, however, has shown that the single stimulus method is as precise as traditional methods in which the standard is presented on each trial (Morgan et al., 2000). Furthermore, the fact that the present study replicated the previously observed recency effect with sequentially presented size ensembles (Hubert-Wallander & Boynton, 2015) clearly speaks for the method’s validity.

In conclusion, the present study demonstrates that people can extract summary statistics from duration ensembles as well as from size ensembles, and they can do this for simultaneous as well as for sequential ensembles. Nevertheless, the results also indicate clear differences between the two dimensions. When the stimuli of the ensemble are encoded at the same time, mean estimates seem to be biased by domain-specific features, possibly by the interoffset intervals in the case of duration, and by overall ensemble size in the case of size. When the stimuli are presented sequentially, the integration of the information seems to differ between the two dimensions, with a recency effect for size but not for duration.

## Data Availability

The datasets generated during and/or analyzed during the current study are available via OSF (https://osf.io/u3t2j/).

## Footnotes

1 This conclusion is based on the fact that the geometric mean is the exponential transform of the average of the log-transformed time intervals (see Ren et al., 2020).
